# Convergence of heteromodal lexical retrieval in the lateral prefrontal cortex

**DOI:** 10.1038/s41598-021-85802-5

**Published:** 2021-03-18

**Authors:** Alexander A. Aabedi, Sofia Kakaizada, Jacob S. Young, Jasleen Kaur, Olivia Wiese, Claudia Valdivia, Saritha Krishna, Christina Weyer-Jamora, Mitchel S. Berger, Daniel H. Weissman, David Brang, Shawn L. Hervey-Jumper

**Affiliations:** 1 Department of Neurological Surgery, University of California San Francisco, San Francisco, CA 94143 USA; 2 Department of Psychology, University of Michigan At Ann Arbor, Ann Arbor, MI 48109 USA; 3 Department of Psychiatry, Zuckerberg San Francisco General Hospital, San Francisco, CA 94110 USA

**Keywords:** Neuroscience, Cognitive neuroscience, Computational neuroscience, Diseases of the nervous system, Neurology

## Abstract

Lexical retrieval requires selecting and retrieving the most appropriate word from the lexicon to express a desired concept. Few studies have probed lexical retrieval with tasks other than picture naming, and when non-picture naming lexical retrieval tasks have been applied, both convergent and divergent results emerged. The presence of a single construct for auditory and visual processes of lexical retrieval would influence cognitive rehabilitation strategies for patients with aphasia. In this study, we perform support vector regression lesion-symptom mapping using a brain tumor model to test the hypothesis that brain regions specifically involved in lexical retrieval from visual and auditory stimuli represent overlapping neural systems. We find that principal components analysis of language tasks revealed multicollinearity between picture naming, auditory naming, and a validated measure of word finding, implying the existence of redundant cognitive constructs. Nonparametric, multivariate lesion-symptom mapping across participants was used to model accuracies on each of the four language tasks. Lesions within overlapping clusters of 8,333 voxels and 21,512 voxels in the left lateral prefrontal cortex (PFC) were predictive of impaired picture naming and auditory naming, respectively. These data indicate a convergence of heteromodal lexical retrieval within the PFC.

## Introduction

Lexical retrieval, the process by which a word that best conveys a given concept is selected from the lexicon, is universally required for natural language and impaired in nearly all forms of aphasia^1,2^. Models of lexical retrieval during speech production describe two main processing steps. First, the meaning of a word is accessed (i.e., lexical semantics). Next, the sound code is accessed (i.e., lexical phonology)^3–8^. Incongruencies exist in current lexical retrieval models in part because the process has been predominantly examined using visual confrontation (i.e., picture naming) tasks^6,9^. Specifically, various authors have proposed that picture naming begins with visual object recognition, followed by retrieval of nonverbal conceptual knowledge of the stimulus^10^. These visuoperceptual processes then facilitate access to lexical semantics and lexical phonology. To provide a more ecologically valid and comprehensive representation of lexical retrieval, a number of studies have since incorporated non-visual, auditory-only naming tasks^11^. However, rather than generate a unified cognitive model of lexical retrieval that encompasses distinct sensory modalities, studies employing visual and auditory naming tasks have presented conflicting results.

Existing convergent mechanisms of lexical retrieval describe overlapping visual and auditory neural systems within the dominant frontotemporal region. For example, Hamberger et al*.* (2001) used direct electrical stimulation (DES) to show that both visual and auditory naming sites converge within the posterior temporal cortex^12^. Similarly, Hamberger et al*.* (2014) used functional magnetic resonance imaging (fMRI)^13^ to show that frontotemporal regions involved in auditory naming overlapped with those in picture naming. Finally, Forseth et al*.* (2018) used a combination of fMRI, DES, and electrocorticography to demonstrate broad overlapping networks encompassing picture and auditory naming^14^.

Alternatively, divergent mechanisms support distinct anatomic and network correlates of lexical retrieval in response to visual and auditory stimuli. Malow et al*.* (1996) found “auditory only” naming sites primarily in posterior temporal regions using DES^15^. Hamberger et al*.* (2001)^12^ and Hamberger and Seidel (2009)^16^ reported that patients with lesions in the same area had impairments in visual but not auditory naming, and that the anterior temporal lobe conferred specificity for auditory naming, but not picture naming.

Anomic/dynsomic aphasia is a common finding in adult brain tumor patients and the presence or absence of overlapping neural systems for lexical retrieval have tangible consequences for patients^17^. For example, cognitive rehabilitation strategies for patients with aphasia may focus on compensatory strategy training such as paced speech, associative cuing, and verbal circumlocution if separate neural systems exist for visual and auditory stimuli^18–23^. If, however, distinct sensory stimuli converge on a single brain region, cognitive rehabilitation strategies may be better served by environmental interventions, such as supportive communication strategies^24,25^.

Incongruent results among existing studies may reflect limitations of the methodologies used to study heteromodal lexical retrieval. Indeed, DES is restricted to regions exposed during brain mapping surgery, may be affected by the administration of anesthetics, and induces non-physiologic, backward propagation of action potentials, thereby limiting its spatial specificity^26–28^. Furthermore, studies employing DES often do not assess subcortical tissue, differentiate speech arrest (i.e., a transient dysfunction in general speech production) from true anomia, or match stimuli on content category. Finally, while functional imaging modalities such as electrocorticography, fMRI, and PET offer correlational insights with varying temporal and spatial precisions, they cannot generate conclusions about requisite brain areas with causal certainty^29^.

Lesion symptom mapping (LSM) is poised to identify cortical and subcortical regions that are necessary for a given task, either directly or indirectly via involvement of tissues connected to distant brain regions (diaschisis)^30^. Traditionally, LSM was implemented using a chronic stroke lesion model, thereby offering a view of language processing based on vascular territory. However, LSM has since been expanded to include lesions formed by intrinsic brain tumors, cytoreduction surgery, traumatic brain injury, and neurodegenerative disease^31–34^. Because dominant-hemisphere intrinsic brain tumors in particular lead to substantial rates of dysnomic aphasia^35^ and can encompass broad cortical and subcortical regions without confinement to stereotyped vascular distributions, they may serve as optimal lesion models to study lexical retrieval in response to visual and auditory stimuli. Furthermore, ensuing results may inform disease-specific therapeutic strategies during cognitive rehabilitation.

In this study, we used a permutation-based, multivariate approach to LSM to identify cortical and subcortical regions necessary for heteromodal lexical retrieval. Because recent data using a combination of functional imaging and DES offer conflicting models of lexical retrieval, it remains unknown whether separate auditory- and visual-only regions exist, or whether the neural systems subserving lexical retrieval exist through distributed convergence zones. Thus, our aims were twofold: (1) to determine whether lesions in frontotemporal regions impair lexical retrieval in a sensory-specific manner and (2) to identify causal convergence zones with a lesion-symptom mapping framework.

The left lateral prefrontal cortex (PFC) in particular is an excellent anatomic candidate for convergence of lexical retrieval given its dual role in (a) processing lexical semantics (specifically within the pars orbitalis and triangularis) and (b) serving as an interface for working memory and executive control in the presence of competing alternatives^36,37^. Considering the reliance of picture naming and auditory naming on these cognitive functions, and evidence of heteromodal convergence hubs in non-lexical domains^38^, we hypothesize that tumor infiltration of the left lateral PFC will be associated with selective impairments in lexical retrieval regardless of input sensory modality.

## Materials and methods

### Participants

Eighty-nine adult patients presenting with World Health Organization (WHO) II-IV gliomas and brain metastasis at the University of California San Francisco (UCSF) between 2017 and 2020 were recruited in a longitudinal prospective clinical trial of language and neurocognitive outcomes (trial registration: NCI-2020-02,286, 11/30/2017). This included fifty-three adult patients (participants) with dominant hemisphere tumors and a disease- and age-matched control cohort of thirty-six patients (controls) with non-dominant hemisphere tumors. No patients had pre-existing neurological impairment prior to tumor diagnosis. Hemisphere of language dominance was established via magnetoencephalography (MEG)^39^. Tumor histology was classified according to the WHO 2016 classification of CNS tumors^40^. Fresh tissue samples were imaged using stimulated Raman histology with cell counting of core specimens using previously established protocols^41^. Participants and controls were on a standard regimen of dexamethasone and levetiracetam for control of brain edema and seizures, respectively. All patients provided written informed consent for study enrollment, which was approved by UCSF’s institutional review board (CHR-17-23,215). The study was performed in accordance with the Declaration of Helsinki.

### Experimental design and statistical analysis

#### Preoperative language assessments

Each participant and control underwent a language assessment one day prior to cytoreduction surgery using the validated Quick Aphasia Battery (QAB) which provides weighted scores for each of its seven predefined language domains (“subtests”): word comprehension, sentence comprehension, word finding, grammatical construction, motor speech, repetition, and reading^42^. The QAB was used in this study given its prior use in adult brain tumor patients and ability to provide rapid (yet comprehensive) assessments of language functions. Furthermore, the QAB was particularly valuable for the purposes of this study as it provides a validated measure of lexical retrieval through its word finding subtest. Nine participants did not undergo QAB testing and were excluded from components of the analysis that required QAB scores. QAB scores between participants and controls were compared using the two-tailed Wilcoxon rank-sum test after confirming non-normality of scoring distributions both visually and via the Shapiro–Wilk test. This comparison was 80% powered to identify a medium-to-large effect size (Cohen’s *d* of 0.67) using a significance level of 0.05. To identify sources of potential confounding in language scores, categorical comparisons of demographics and baseline clinical data between study participants and controls were performed with Pearson’s chi-squared test.

In addition to completing the QAB, each of the fifty-three participants performed four additional language tasks for use in lesion-symptom mapping. These tasks consisted of naming pictorial representations of common objects and animals (Picture Naming, PN), reading two-syllable text (Text Reading, TR), naming common objects and animals via auditory descriptions (Auditory Naming, AN) and describing line drawings with intact syntax (Syntax, Syn)^28,43^. The correct answers for all four tasks were matched on word frequency (i.e., commonality within the English language) using SUBTLEX_WF_ scores provided by the Elixcon project (http://elexicon.wustl.edu/) and on content category^44^.

All of the tasks were delivered on a laptop with a 15-inch monitor (60 Hz refresh rate) that was positioned two feet away from the seated patient in a quiet clinical setting. Task stimuli were randomized and presented using PsychToolbox^45,46^. Slides were manually advanced by the research coordinator either immediately after the participant provided a response or after six seconds if no response was given. All of the tasks were scored on a scale from 0 to 4 by a trained clinical research coordinator who was initially blinded to all clinical data (including imaging studies) using the guidelines provided by the QAB. No participants had uncorrectable visual or hearing loss.

To identify which language tasks were most strongly associated with the validated measure of lexical retrieval provided by the QAB (i.e., the word finding subtest), generalized linear models were fitted between the word finding subtest and each of the four language tasks. Model significance was determined using a two-tailed F-test. Principal components analysis (PCA) was performed to collapse the five language measures into a smaller set of dimensions (i.e., principal components) that account for a majority of the variance in the dataset. By removing redundancies in the behavioral data, PCA is able to identify common cognitive constructs. In this study, the largest resulting principal component (i.e., defined by a weighted combination of our five language measures) was used in lesion-symptom mapping to identify brain regions subserving a common cognitive construct. All statistical analyses were performed on R version 3.6.2. Corrections for multiple comparisons among the language data were made by controlling the family-wise error rate with the Holm-Bonferroni method^47^.

#### Magnetic resonance imaging and pre-processing

Each participant underwent a standard preoperative imaging protocol on a 3 T scanner including a) pre- and post-contrast T1-weighted imaging with gadolinium and b) T2-weighted fluid attenuation inversion recovery (FLAIR) imaging with slice thicknesses between 1 and 1.5 mm^43^. All imaging was performed within three days of the language assessments.

Lesions were segmented either manually or semi-automatically using ITK-SNAP 3.8.0 (http://www.itksnap.org/) by a trained co-author blinded to language outcomes scores (AAA)^48^. The borders of contrast enhancement on T1-weighted post-gadolinium sequences were used to identify lesion boundaries in patients with WHO grade IV contrast enhancing tumors (n = 43 patients). Abnormal FLAIR signal was used to identify lesion boundaries in patients with WHO grades II and III non-enhancing tumors (n = 10)^32,49^. Accuracy of the lesion masks was independently confirmed by a separate examiner also blinded to language outcomes scores (SHJ) and disagreements were resolved by reaching a consensus.

Using Clinical Toolbox on SPM12 (https://www.fil.ion.ucl.ac.uk/spm/software/spm12/), each anatomical T1-weighted image was normalized to the standardized Montreal Neurological Institute template (MNI152)^50,51^. The resulting transformation matrix was applied to the participant’s corresponding lesion mask to facilitate comparisons across participants. For participants with non-enhancing lesions, the T2-weighted FLAIR derived lesion mask was registered to the anatomical image prior to normalization.

#### Lesion-Symptom and Statistical Analysis

Support vector regression lesion-symptom mapping (SVR-LSM) was performed to identify the precise anatomic locations associated with lower scores on each of the four language tasks (PN, TR, AN, and Syn) and the first principal component derived from PCA^52^. Analyses were conducted using the SVRLSMgui package (https://github.com/atdemarco/svrlsmgui/) for MATLAB which provides a permutation-based multivariate approach to lesion-symptom mapping with significant advantages over mass-univariate analyses^53^. As opposed to traditional voxel lesion-symptom mapping (VLSM), by including every lesion voxel as a simultaneous covariate, SVR-LSM accounts for the inherent spatial autocorrelation of brain tumors^54,55^. Additionally, by performing random permutations of the behavioral data to create null distributions, it is robust to the Type I and II errors that often arise from false discovery rate and Bonferroni methods for multiple comparisons corrections, respectively^56^.

Total lesion volume was linearly regressed out of both the lesion masks and language scores to limit the biasing impact of large lesions on language impairment, while retaining sensitivity to fluctuations in task performance. For inclusion in each task-based analysis, each voxel was required to include at least two participants with overlapping lesions^57^. A threshold of *P* < 0.005 was used to determine statistical significance for individual voxels while a threshold of *P* < 0.05 was used for cluster-level (i.e., groups of contiguous voxels) corrections after performing 10,000 permutations of the language data across participants. Hyperparameters were left at their default settings (gamma of 5 and cost of 30). Analyses were fully parallelized on a high-performance computer with 32 cores at 4.2 GHz and 256 GB of random-access memory. SPM12 was used to generate statistical parametric maps and BrainNet Viewer (https://www.nitrc.org/projects/bnv/) to generate three-dimensional representations of the resulting statistical maps^58^.

## Results

**Figure 1:**
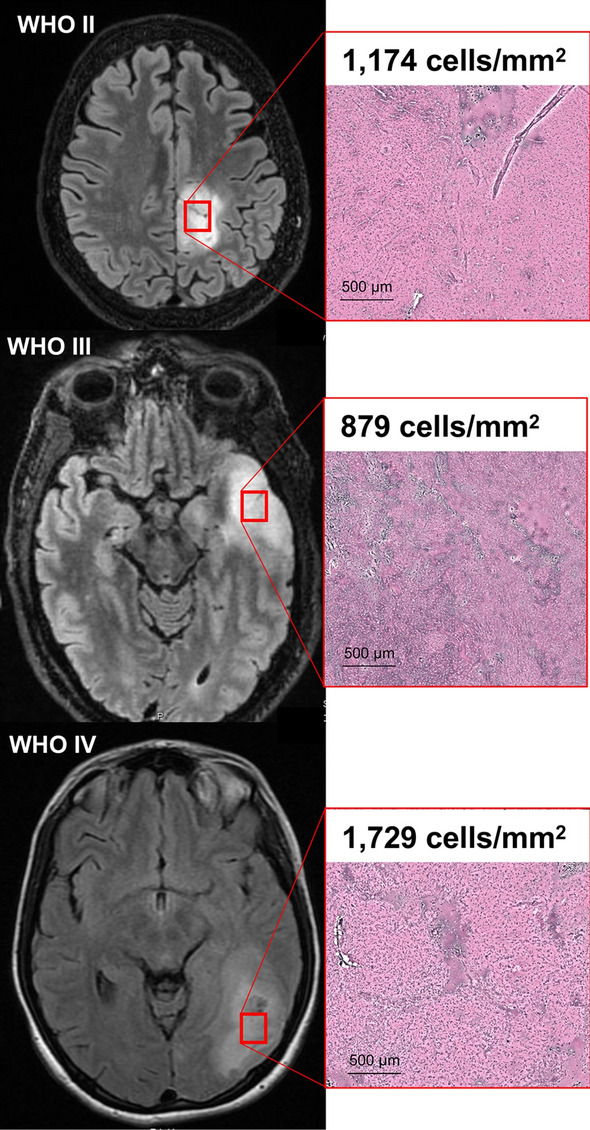
T2-weighted fluid attenuation inversion recovery (FLAIR) images of three participants with World Health Organization (WHO) grade II, III, and IV glioma (left). Red boxes indicate regions of FLAIR signal abnormality biopsied for pathologic examination and cell-counting with Raman scattering microscopy (right). Pseudo-H&E images of fresh biopsy specimens demonstrate ablation of normal cytoarchitecture across all WHO tumor grades. Here, lesion cellularity and necrotic features escalate with increasing WHO grade.

Demographics and clinical data are summarized in Table 1. There were no significant differences in mean age, handedness, education, or oncologic features between participants and controls. Participants were significantly more likely to be male and to have left-sided tumors. Tissues sampled from regions of FLAIR or T1-weighted post-gadolinium signal abnormality from three different participants with WHO grade II, III and IV gliomas are presented in Fig. 1. All participants and controls had WHO II-IV gliomas as defined by WHO 2016 molecular subclassifications and their imaged tissues demonstrated cellular neoplasms with extensive disruption of normal cytoarchitecture.

**Table 1:** Demographics and clinical summary.

Characteristic	Participants	Controls	*P*-value
*n*	53	36	
Sex	17 F, 36 M	22 F, 14 M	0.013
Mean Age (SD)	51.2 (17.3)	49.4 (15.0)	0.54
Handedness	3 L, 47 R, 3 unk	6 L, 27 R, 3 unk	0.19
Education			0.35
High school	10	6	
Some college	11	7	
Bachelors	11	13	
Graduate	6	4	
Unk	15	6	
Tumor Laterality	49 L, 4 R	1 L, 35 R	3.6 × 10^–16^
Tumor Grade/Pathology			0.18
WHO Grade I—II	11	9	
WHO Grade III	15	4	
WHO Grade IV	24	23	
Metastasis	3	0	

Comparisons between categorical variables were made with the chi-squared test while continuous variables were compared with the Wilcoxon rank-sum test for participants with dominant hemisphere lesions and controls with non-dominant hemisphere lesions. Handedness was determined using preoperative magnetoencephalography. Education was self-reported in patients who completed the Neuro-QoL assessment. Pathologic diagnoses were made by board-certified pathologists using the World Health Organization (WHO) Revised Classification of Tumors in the CNS. Unk.-Unknown.

**Figure 2:**
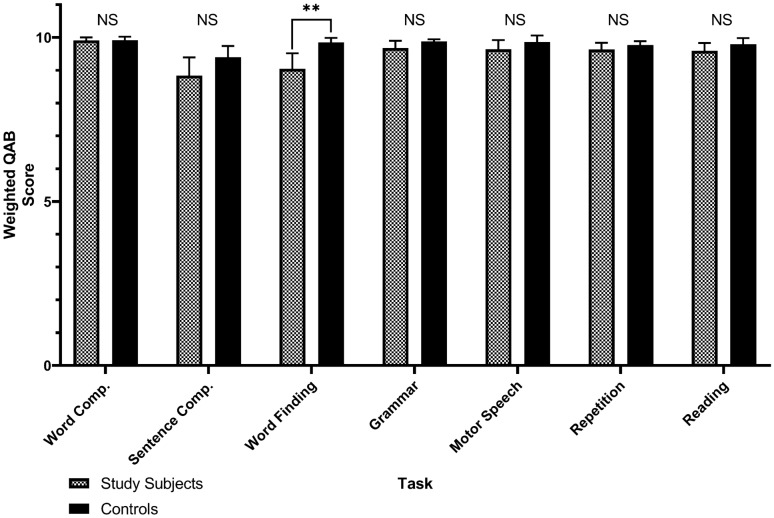
Pairwise Wilcoxon rank-sum tests between 44 patients with dominant-hemisphere intraparenchymal tumors (participants) and 36 patients with non-dominant tumors (controls) on each of the seven predefined Quick Aphasia Battery (QAB) subtests. **P = 0.0096, corrected for multiple comparisons with the Holm-Bonferroni method. NS-not statistically significant (*P* > 0.05). Word Comp.-word comprehension. Sentence Comp.-sentence comprehension. Grammar-grammatical construction. Error bars represent standard error.

**Figure 3:**
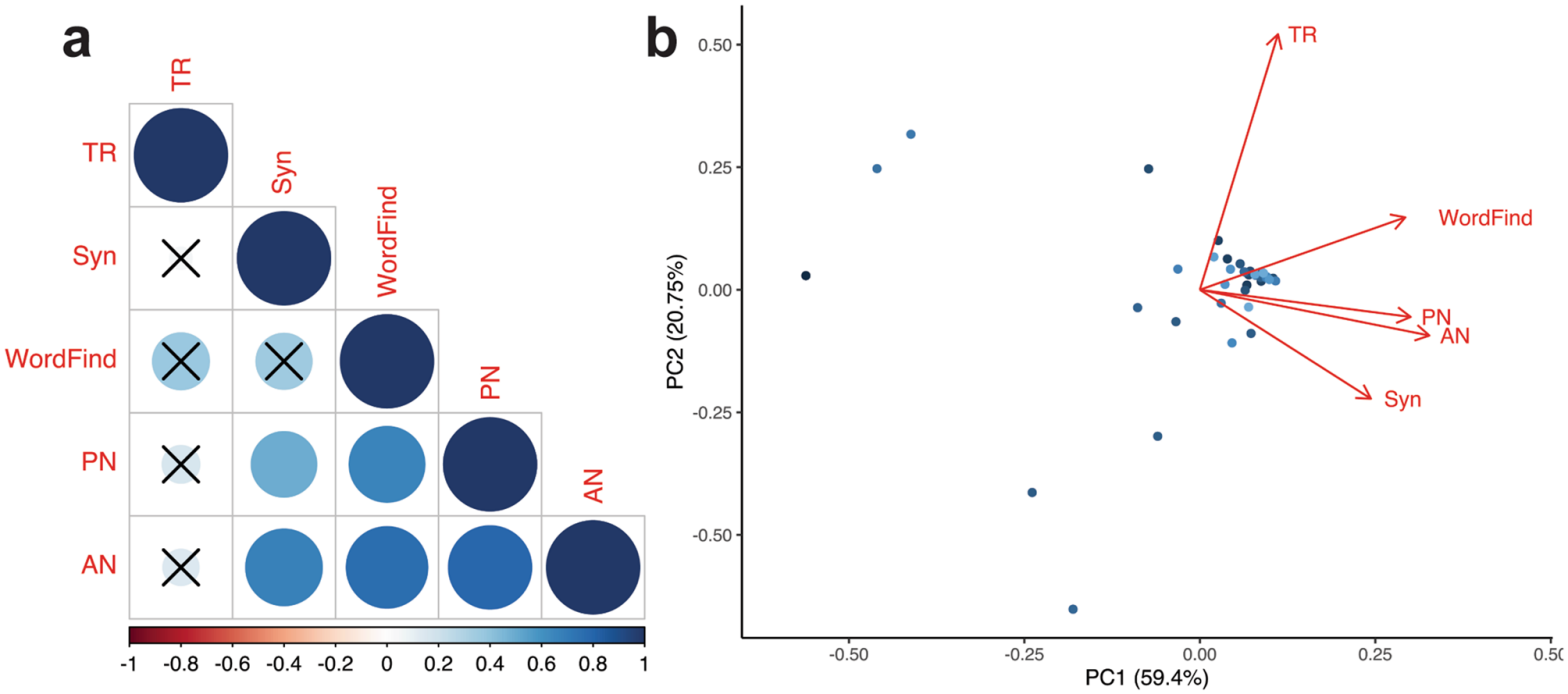
Operationalization of the word finding subtest of the QAB (WordFind) using four computerized language tasks: picture naming (PN), text reading (TR), auditory naming (AN), and syntax formation (Syn). a correlogram summarizing the results of univariate generalized linear models fitted to each language task and WordFind. Color bar represents each correlation coefficient and crosses indicate non-significant associations (*P* > 0.05) after corrections for multiple comparisons. b biplot between principal component 1 and 2 (PC1 and PC2) derived from principal components analysis (PCA) on WordFind and the four language tasks. Corresponding loadings are represented by the red arrows.

Our main goal was to identify the neuroanatomical regions that are necessary for heteromodal lexical retrieval using a lesion-symptom model. As such, first, we compared language domain performance between participants and controls across all seven QAB subtests to establish whether or not participants had significant impairments in lexical retrieval. Compared to controls, participants exhibited statistically significantly lower performance on word finding (*P* = 0.0096), but not on any of the six remaining subtests of the QAB (Fig. 2). Second, we sought to identify associations between each of the four language tasks (Table 2) and the word finding subtest using generalized linear models computed across all participants (Fig. 3a). Word finding was predicted by picture naming (*r* = 0.66, *P* = 0.000029) and auditory naming (*r* = 0.76, *P* = 0.0000001) but not by text reading (*r* = 0.37, *P* = 0.093) or Syn (*r* = 0.36, *P* = 0.093). Significant associations were also found between PN and AN (*r* = 0.80, *P* = 0.000000008), Syn and PN (*r* = 0.49, *P* = 0.0088), and Syn and AN (*r* = 0.68, *P* = 0.000016).

**Table 2:** Language testing.

Task (*n* trials)	Mean Score	SE Mean
Picture Naming (48)	3.84	0.051
Text Reading (27)	3.97	0.014
Auditory Naming (32)	3.62	0.074
Syntax (28)	3.80	0.058

Tasks were delivered in a quiet clinical setting and matched on word frequency. Each stimulus was scored from 0 to 4 using guidelines provided by the Quick Aphasia Battery.

Given these results, we next performed PCA to a) reveal and remove redundancies in the behavioral data and b) identify a set of weighted combinations of the language measures that subsequently represent a single, common cognitive construct. PCA revealed two principal components (PC), PC1 and PC2, that were responsible for 59.4% and 20.75% of the variance in the five language measures, respectively (Fig. 3b). Picture naming, auditory naming, and word finding demonstrate the greatest collinearity with PC1. Text reading, on the other hand, is roughly collinear with PC2.

**Figure 4:**
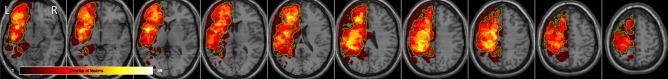
Map depicting regions in which at least two participants had overlapping lesions, projected on the standard-space Montreal Neurological Institute template (MNI152), total *n* = 48. Regions in which actual overlap occurred and where the analysis was restricted to is outlined in green. Accordingly, five participants with non-overlapping right-sided tumors were excluded from lesion-symptom mapping.

Having established the importance of picture naming and auditory naming in explaining the variance in the word finding subtest, we then used these two tasks in conjunction with SVR-LSM to uncover the anatomical regions necessary for visually- and auditorily-prompted lexical retrieval, respectively. Of the 53 participants, 48 had lesions with at least one overlapping voxel with another participant and were therefore included in the lesion-symptom mapping analysis. All five excluded participants had right-sided, dominant-hemispheric tumors (confirmed by MEG) with non-overlapping lesions. The final lesion overlap map used for SVR-LSM is outlined in green in Fig. 4.

**Figure 5:**
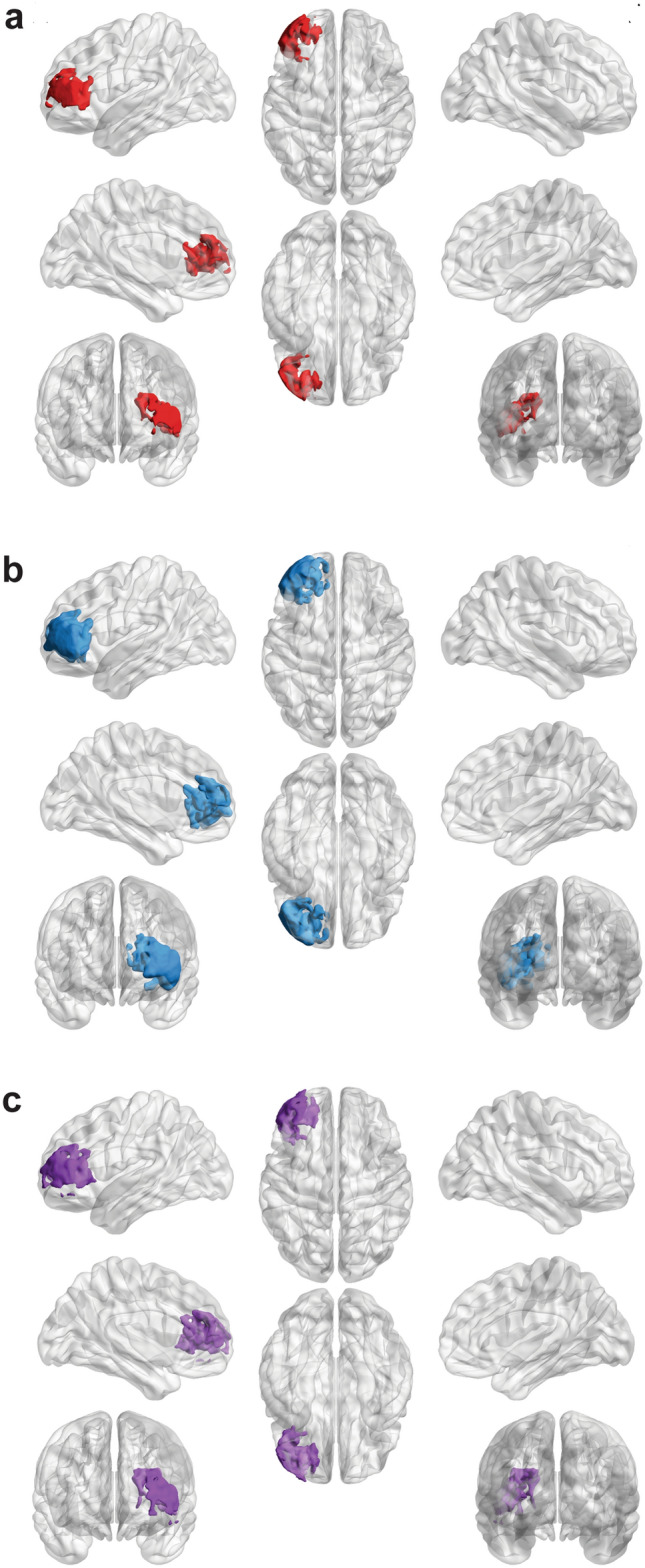
Results of support vector regression lesion-symptom mapping (SVR-LSM) after voxels thresholded at *P* < 0.005 underwent cluster-level corrections with 10,000 permutations (cluster threshold = *P* < 0.05). 3-Dimensional models of resulting clusters were generated using the Montreal Neurological Institute template via BrainNet Viewer (https://www.nitrc.org/projects/bnv/). a Lesions in a cluster of 8,333 voxels in the left lateral PFC were predictive of impaired picture naming (*P* = 0.045). b Lesions in a larger cluster of 21,512 were voxels predictive of impaired auditory naming (*P* = 0.0034). Clusters in a and b overlap completely with the cluster in a extending deeper into subcortical regions. c Results of SVR-LSM using the principal component scores for each participant on the first principal component (PC1). This cluster consists of 11,944 voxels (*P* = 0.019) and demonstrates overlap with the clusters in a and b.

Using this lesion map, SVR-LSM was then performed for each of the four language tasks testing the hypothesis that lesioned voxels are associated with lower task accuracies. For picture naming, one of eight clusters survived cluster-level corrections (8,333 voxels, *P* = 0.045). The resulting cluster for picture naming is centered in the left lateral PFC and encompasses Brodmann areas 10 and 45–48 (Fig. 5a). For auditory naming, one of ten clusters survived thresholding in an analogous region of the lateral PFC (Fig. 5b), except with greater involvement of subcortical areas (21,512 voxels, *P* = 0.0034). Notably, no significant clusters for auditory naming were observed in the temporal areas. The significant cluster in picture naming overlaps completely with, and accounts for 38.7% of, auditory naming’s significant cluster. Finally, SVR-LSM was performed using PC1 from the PCA. Only one of fourteen clusters survived cluster-level corrections (11,944 voxels, *P* = 0.019). This cluster overlaps with the resulting clusters from picture naming and auditory naming (Fig. 5c).

Neither TR nor Syn survived cluster-level corrections. For TR, no significant voxels were found after voxel-based thresholding. Syn, on the other hand, yielded smaller clusters of statistically significant voxels interspersed throughout the left anterior frontal lobe. However, the size of these clusters fell short of the critical threshold size that was derived from performing SVR-LSM on random permutations of the scores.

## Discussion

Cognitive models of lexical retrieval have been heavily influenced by visual confrontation (picture naming) tasks. Further, existing studies have implemented methods that either lack causal certainty or precise spatial localization. Therefore, published results are mixed with respect to whether visual and auditory stimuli represent overlapping (convergent) or non-overlapping (divergent) neural systems. This unresolved conflict has direct implications on cognitive rehabilitation strategies for brain tumor patients with dysnomic aphasia. In this study, we propose a convergent model of lexical retrieval within a lesion-symptom framework for both visual and auditory inputs. First, we established that intrinsic brain tumors cause extensive disruption of normal cytoarchitecture (Fig. 1), leading to selective impairments in word finding (Fig. 2). This finding is in line with previous reports of the prevalence of selective dysnomia in patients with dominant hemisphere intrinsic brain tumors^59^. Furthermore, it supports the use of our brain tumor lesion model in particular to study lexical retrieval, since participants in other clinical populations tend to have additional confounding language impairments^60,61^.

Next, we demonstrated strong correlations between accuracy on picture naming and auditory naming, providing initial evidence of the existence of lexical retrieval pathways that are agnostic to the sensory modality of the input (Fig. 3). From a behavioral perspective, this finding replicates those in lesion studies performed in several clinical populations. For instance, Hamberger and Seidel (2003) found that patients with left temporal lobe epilepsy had co-occurring impairments in visual and auditory naming compared to a) healthy controls and b) patients with right temporal lobe epilepsy^62^. Miller et al. (2010) and Hirsch et al*.* (2016) also reported significant associations between picture naming and auditory naming in lesion studies of patients with dementia^63,64^. Hirsch et al*.* in particular used PCA in a cohort of 458 patients to reveal a unique redundancy between picture naming and auditory naming that was not shared with any of their other twenty-five cognitive and linguistic measures. In the present study, PCA led to analogous results: the similarity in loadings between PN, AN, and word finding argue for a single linguistic construct (i.e., lexical retrieval) that may be differentiated from constructs contributing to other linguistic measures such as TR and Syn.

As predicted by the results of our language assessments, multivariate lesion-symptom mapping revealed overlapping clusters for picture naming, auditory naming, and PC1 in the left lateral prefrontal cortex (Fig. 5A-C). The significance of this brain region in particular is that it is capable of processing the critical components of lexical retrieval, namely semantic access and executive control (or more specifically, selection between competing items). Indeed, various authors have proposed that the lateral PFC serves as a generic region for multimodal integration given its preferential activation during demanding tasks that require selection between competing alternatives which may include both semantic and phonological items^65,66^. Future investigation will be required to dissociate the contributions of the lateral PFC to these interdependent components.

Notably, in contrast to prior DES studies^12,67^, a separate cluster specific to auditory naming was not identified in the anterior temporal lobe, a well-represented region in our lesion overlap mask. Our results instead align with a number of more recent, mixed-method studies that argue for a convergence hub in the lateral PFC for both visual and auditory inputs^14,68^. Specifically, by timing the evolution of cortical responses to the onset of task stimuli using electrocorticography, Forseth et al*.* (2018) showed that the inferior frontal gyrus (a component of the lateral PFC) serves as an interface between lexical and phonological pathways during both picture and auditory naming^14^. Taken with the results of the present lesion study, these findings provide compelling and causal evidence that lexical retrieval indeed represents a unified cognitive construct that is agnostic to the input modality. The lack of convergence of TR and Syn in these regions, despite sharing the same input modality as PN, further argues that lexical retrieval predominantly relies on downstream language networks that are anatomically distinct from those engaged during orthographic (TR) and syntactic (Syn) processing. For example, in contrast to our cohort of patients with anomic aphasia, individuals with acquired phonological alexia (i.e., impaired text reading) demonstrate global impairments in phonological processing that generalize to non-visual sensory modalities, but typically have intact lexical-semantic processing^69^.

Notably, case reports and small clinical series of patients with modality-specific naming impairments such as visual (i.e. optic aphasia), auditory, and tactical anomia from brain lesions may seem to argue against the existence of a single construct subserving lexical retrieval^70–72^. However, these studies have not been widely validated in large clinical cohorts using rigorous image-based methods and thus cannot be adequately evaluated for confounding variables. Specifically, concurrent damage to primary sensory processing or association areas cannot be ruled out without the granularity provided by voxel-based analyses. For instance, Hamberger and Seidel (2009) reported that a cohort of fourteen patients with anterior temporal lobe lesions (defined as < 5 cm from the temporal pole) had impaired auditory, but not picture naming^16^. However, of those fourteen patients, only five had structural lesions. The remaining “anterior lesion” patients were defined by the presence of seizure foci on subdural or scalp EEG. Given a) the absence of lesion overlap maps for examination and b) the ability of epileptic foci to transiently impair remote brain areas, we cannot rule out the possibility that the observed differences in auditory and picture naming performance were due to confounding damage to auditory sensory processing areas (i.e. A1-A3). Indeed, the “auditory-only” naming sites identified by Malow et al*.* (1996) via DES (i.e. transient induction of iatrogenic lesions) are chiefly located in the superior temporal gyrus where various features of auditory stimuli are encoded^73–75^. Analogous concerns have also been raised about other modality-specific anomia syndromes^76^.

Since the late nineteenth century, it has been known that the brain, at its most fundamental level, is made of individual cells which together form discrete networks that influence cognition and behavior. Efforts to uncover the role of neuroanatomic structures and underlying network dynamics have established both causal and correlative cognitive frameworks. However, these physiological models may lack disease-specific relevance. Therefore, the discovery of overlapping neural systems for lexical retrieval in a brain tumor lesion-symptom mapping study has implications beyond causal adjudication between established scientific models. One salient example comes from competing rehabilitative strategies for patients with aphasia. On the one hand, compensatory training relies on the use of alternative stimuli through strategies such as paced speech, associative cuing, and verbal circumlocution to leverage distant yet overlapping neural systems for a given sensory domain. Compensatory training may therefore be considered most promising in the setting of divergent visual and auditory lexical retrieval systems^18–23^. If, however, distinct sensory stimuli converge on a single brain region and/or network, it may be best if cognitive rehabilitation strategies focus on environmental interventions, such as supportive communication strategies following a lesion to that region^24,25^. The optimal rehabilitation strategy for patients with brain tumors and subsequent aphasia continues to be an area of active investigation. Therefore, causal evidence using disease-specific models of physiology will contribute to a nuanced understanding of therapeutic options.

### Limitations of the Present Study

It is first worth discussing the typical limitations that apply to any lesion-symptom analysis, namely that conclusions about behavioral contributions of brain areas lying outside our lesion masks cannot be made. For this reason, we expanded our analysis to include voxels in which at least two patients overlapped, rather than more restrictive cutoffs of five or more found in previous studies. By performing multivariate comparisons on a high-performance computer that can support these increasingly memory- and computationally intensive analyses, we were able to avoid the reductions in statistical power that typically constrain studies employing mass-univariate tests with post-hoc corrections for multiple comparisons. While lowering the lesion overlap threshold may increase exposure to outlier effects, our implementation of non-parametric statistics for lesion-symptom mapping mitigate this concern by calculating exact p-values without any a priori assumptions about the underlying distribution. In our study, as in others, the impact of outliers was greatly attenuated by performing 10,000 random permutations of the input behavioral data to generate a null distribution for each test statistic^77^. Despite these measures, however, our lesion mask was not able to capture posteroinferior regions of the perisylvian network, thereby restricting our conclusions to the frontal cortex, anterior temporal cortex, and angular gyrus.

Second, the expansive nature of the lesions under study (which inherently facilitates group-level analyses) simultaneously limits our ability to precisely localize the neuronal subpopulations involved in each of the two convergent tasks or determine how these subpopulations may causally interact to produce correct responses.

Furthermore, while there is some debate on whether brain tumors can serve as reliable clinical models for lesion-symptom analyses, this position can be extended to stroke for which VLSM was developed and is most commonly implemented. Furthermore, brain tumor histology confirmed disordered neural structures, increased cellularity, and altered cytoarchitecture (Fig. 1). Because perfect clinical models for brain lesions likely do not exist, the literature may be best served by an increase, rather than a decrease, in the diversity of the clinical populations under study^78^.

## Conclusion

To summarize, generalized linear modeling and principal components analysis revealed multicollinearity between picture naming, auditory naming, and word finding tasks. Support vector regression lesion-symptom mapping across participants was used to uncover associations between lower task accuracies on each of the four language tasks and lesioned voxels. Picture naming and auditory naming lesions demonstrated overlapping clusters within the left lateral PFC. This study demonstrates that cortical and subcortical brain regions involved in lexical retrieval from visual and auditory stimuli represent overlapping neural systems.
